# Endometrial Microbiome and Reproductive Receptivity: Diverse Perspectives

**DOI:** 10.3390/ijms262110796

**Published:** 2025-11-06

**Authors:** Galina Stoyancheva, Nikolina Mihaylova, Maria Gerginova, Ekaterina Krumova

**Affiliations:** 1 Department of Microbial Genetics, The Stephan Angeloff Institute of Microbiology, Bulgarian Academy of Sciences, 1113 Sofia, Bulgaria; mariagg@microbio.bas.bg; 2 Department of Immunology, The Stephan Angeloff Institute of Microbiology, Bulgarian Academy of Sciences, 1113 Sofia, Bulgaria; mihaylova_n@microbio.bas.bg; 3 Department of Mycology, The Stephan Angeloff Institute of Microbiology, Bulgarian Academy of Sciences, 1113 Sofia, Bulgaria

**Keywords:** endometrial microbiome, *Lactobacillus*, immunological mechanisms, 3D human endometrial culture models, oxidative stress, antioxidant activity of lactobacilli

## Abstract

The human endometrium, previously considered a sterile environment, is now recognized as a low-biomass but biologically active microbial niche critical to reproductive health. Advances in sequencing technologies, particularly shotgun metagenomics, have provided unprecedented insights into the taxonomic and functional complexity of the endometrial microbiome. While 16S rRNA sequencing has delineated the distinction between *Lactobacillus*-dominant and non-dominant microbial communities, shotgun metagenomics has revealed additional diversity at the species and strain level, uncovering microbial signatures that remain undetected by amplicon-based approaches. Current evidence supports the association of *Lactobacillus* dominance with endometrial homeostasis and favorable reproductive outcomes. Dysbiosis, characterized by increased microbial diversity and enrichment of anaerobic taxa such as *Gardnerella*, *Atopobium*, *Prevotella*, and *Streptococcus*, is linked to chronic endometritis, implantation failure, and adverse IVF results. Beyond compositional differences, the endometrial microbiome interacts with the host through immunological, metabolic, and epigenetic mechanisms. These interactions modulate cytokine signaling, epithelial barrier integrity, and receptivity-associated gene expression, ultimately influencing embryo implantation. However, discrepancies between published studies reflect the lack of standardized protocols for sampling, DNA extraction, and bioinformatic analysis, as well as the inherent challenges of studying low-biomass environments. Factors such as geography, ethnicity, hormonal status, and antibiotic exposure further contribute to interindividual variability. Culturomics approaches complement sequencing by enabling the isolation of viable bacterial strains, offering perspectives for microbiome-based biotherapeutics. Emerging 3D endometrial models provide additional tools to dissect microbiome–host interactions under controlled conditions. Taken together, the growing body of data highlights the potential of endometrial microbiome profiling as a biomarker for reproductive success and as a target for personalized interventions. Future research should focus on integrating multi-omics approaches and functional analyses to establish causal relationships and translate findings into clinical practice. This review gives a new insight into current knowledge on the uterine microbiome and its impact on implantation success, analyzed through the lenses of microbiology, immunology, and oxidative stress.

## 1. Introduction

The endometrium is the lining of the uterine cavity. The endometrium, a target tissue for sex steroid hormones, consists of two main structural components—the endometrial glands and the specialized endometrial stroma. As part of their physiological function, these two elements proliferate and grow under the influence of ovarian estrogens (first half of the menstrual cycle). Proliferation is stopped by the antagonistic effect of progesterone, which induces endometrial maturation and differentiation (second half of the menstrual cycle). The endometrial microbiome is the presence, quantities, and diversity of different types of microorganisms in the uterus.

A long-time uterine cavity is considered a sterile environment, free from microorganisms, which provides optimal conditions for implantation and development of the embryo. This concept is based basically on methods for microbiological analysis used in the 20th century, when, through the cultivation techniques, no bacterial colonization in the uterus was detected. In the late 1990s and early 2000s, there were isolated culture studies in which bacteria were accidentally isolated from the endometrium or placenta (e.g., during cesarean sections), but these were considered “contamination” [1,2,3,4]. The real turning point came after 2015, when genetic methods obtained data showing that the presence of microbes in the uterus is real and systematic. With the introduction of molecular methods, including 16S rRNA sequencing and later metagenomics, it became possible to discover microorganisms even in low-biomass sites such as the endometrium [5,6,7,8]. Historically, research in this area is tracked in Table 1. The accumulated data changed the paradigm, showing that the endometrium of healthy women can contain diversity from bacteria, often dominated by *Lactobacillus* spp. This new perspective opens the field for research on the potential role of the microbiome in reproductive health and pathology, including implantation failure, chronic endometritis, and endometriosis [9,10]. According to available data, the rate of colonization of the uterine cavity is relatively low compared to the vagina and cervix [11].

Today, scientific research related to the endometrial microbiome is the focus of many researchers for several key reasons: **(1) Influence on fertility:** The endometrial microbiome probably plays a critical role in the successful implantation of the embryo and maintenance of pregnancy. Some studies show that a certain microbial composition in the endometrium can increase or decrease the chances of successful pregnancy, including in vitro fertilization (IVF). **(2) Linkage with chronic diseases:** Changes in the composition of the endometrial microbiome are related to various chronic gynecological conditions, such as endometriosis and chronic endometritis. Knowledge of the microbiome can help with a better understanding and treatment of these states. **(3) Diagnostics and personalized treatment:** The analysis of the endometrial microbiome can be used as a diagnostic tool to identify specific abnormalities associated with reproductive health problems. This opens the doors for personalized medicine, where the treatment is adapted to the individual microbiome profile of the patient. **(4) Prevention of infections:** Imbalances in the microbiome on the endometrium can increase the risk of infections, such as bacterial vaginosis or infectious endometritis. Understanding the microbiome may help in the development of preventive strategies. **(5) Role in the immune system answer:** The endometrial microbiome can influence local immune response in the uterus, which is important for the protection against pathogens and for toleration of pregnancy.

The research in this area has the potential to revolutionize the approach to diagnostics, treatment, and prevention of different gynecological conditions, such as this one, in a way that significantly improves the quality of life and reproductive outcomes in women. The purpose of this review is a critical analysis of available data and a delineation of the future of juices.

## 2. Methodological Features and Challenges in Endometrial Research Microbiome

### 2.1. Taking on Samples: Risk from Contamination (Cervical/Vaginal Transmission)

The sampling method is quite variable across studies, as different endometrial samples (swabs, biopsies, or aspirates) may be obtained during embryo transfer, hysterectomy, or by transcervical uterine sampling using uterine manipulators and cervical dilators, which could further contribute to cross-contamination with the cervical microbiota [25]. During sample collection, the possibility of contamination is a major obstacle to determining a “baseline microbiome”, especially when studying a low-biomass environment such as the uterus. It is highly likely that a normal endometrial microbiome exists, although it may be at very low levels, making it susceptible to contamination during sampling.

### 2.2. Size of the Study Group and Lack of Healthy Controls

Another key limitation of these studies is the cohort size, strict inclusion and exclusion criteria, and the lack of healthy controls, partly due to the challenges in recruiting patients and the technical challenges of obtaining uterine samples [26].

### 2.3. Low Biomass on the Endometrial Microbiome

The extent of bacterial presence in the uterus is estimated to be between 100 and 10,000 times less than the vaginal microbiome [5,12,27]. When investigating sites with low bacterial abundance using highly sensitive methods, the risk of amplification of contaminating DNA sequences increases, which can significantly alter the results and lead to misinterpretation.

### 2.4. Methods for Research

Conventional methods for microbial detection are not suitable for identifying bacteria that are difficult to culture. Sequencing provides an easier, faster, and more informative analysis of bacterial populations. The emergence of new sequencing technologies, such as next-generation sequencing (NGS), has enabled a much more global assessment of the bacterial composition of the uterus than has been achieved with culture-dependent methods alone. NGS enables species-level quantification using the variable regions of the 16S rRNA gene, potentially allowing the determination of the full composition of the uterine microbiome [26,28,29]. But obtaining precise results requires the availability of negative control samples, standardization of 16S amplicons, and bioinformatic analysis [30]. A significant challenge is that different researchers use different regions of the 16S rRNA gene (V1–V2, V3–V4, V4–V5), which leads to variations in taxonomic resolution. Among the main ones, analytical shotgun metagenomics is also an approach that gives more detailed information for taxonomic and functional profiles, but it is more resource-intensive [31]. In some studies, it also applies qPCR panels for directed research on certain bacterial taxa, which, however, do not cover the whole microbial diversity. Table 2 compares the methods for endometrial microbiome analysis.

### 2.5. Lack of Standardization → Difficulty in Comparing Results

The absence of unified standardization in protocols for sampling, DNA extraction, and bioinformatics analysis significantly makes it difficult to compare results between different research groups and creates uncertainty regarding the clinical applicability of data. Molina et al., 2021 [31] created methodological considerations and recommendations for good practice when analyzing the endometrial microbiome.

## 3. Composition and Dynamics of the Endometrial Microbiome: Is There a Connection with Reproductive Health?

In recent years, numerous scientific studies have been reported to determine the composition of the uterine microbiome. The data obtained are not unambiguous, and the definition of a “normal uterine microbiome” is still a subject of discussion. Many researchers discuss the relationship between the bacterial communities in the uterus and the presence of a receptive, fertile endometrium; the course of normal or pathological pregnancy; and the development of uterine tumors.

In recent years, researchers involved in reproduction have focused on the microbiome at different sites of the genital tract to study its role in the pathogenesis, prevention, and treatment of diseases.

Fertility disorders are the driving force behind numerous studies in gynecology and reproductive medicine. The effect of the female genital microflora on the ability to conceive is still unclear due to insufficient and contradictory data. However, it seems that a flora dominated by *Lactobacillus* spp. plays a key role in determining fertility [32]. Toson et al. [33] recently proposed that the physiological endometrial microbiota should be considered as a group of microorganisms that allows embryo implantation and the birth of a live fetus, regardless of the minimal presence of pathogenic bacteria [33]. Previous studies have defined the normal uterine microbiome in women of childbearing age, where *Lactobacillus* spp. plays a special role [5]. It is currently difficult to define a consensus “healthy” or “core” uterine microbiota. However, some generalizations can be made from the existing data. The most abundant bacteria consistently belong to the following phyla: *Firmicutes*, *Bacteroidetes*, *Proteobacteria*, and *Actinobacteria* [12,26,34]. Within the *Firmicutes*, the genus *Lactobacillus* is a very important component in the majority of studies on the uterine microbiome. Other types of bacteria commonly found in endometrial/uterine swabs include *Bifidobacterium*, *Gardnerella*, *Prevotella*, and *Streptococcus* [6]. Subsequently, abnormal endometrial microbiota is associated with implantation failure, pregnancy loss, and other gynecological and obstetric conditions [35].

Moreno’s team (2016) [6] conducted a comparative study between the endometrial and vaginal microbiota and investigated the functional impact of the composition of the endometrial microbiota on reproductive outcome in patients undergoing in vitro fertilization (IVF). The results suggest that the endometrial and vaginal microbiota may differ in structure and composition in some women. Regarding the endometrial microbiota, all patients were grouped into two groups: the *Lactobacillus* dominant (LD) group and the non-*Lactobacillus* dominant (NLD) group. Analysis of the resulting microbiota reflected significant differences in bacterial diversity, with the NLD group showing greater species diversity than the LD group. In contrast to those with LD microbiota, subjects with NLD microbiota had significantly lower implantation rates (60.7% vs. 23.1%), pregnancy rates (70.6% vs. 33.3%), ongoing pregnancy (58.8% vs. 13.3%), and live birth rates (58.8% vs. 6.7%), as well as higher rates of spontaneous abortion. This adverse effect on pregnancy outcomes was more evident in subjects with high percentages of bacteria from the genera *Gardnerella* and *Streptococcus*.

Kyono and colleagues (2019) [13] apply 16S sequencing in a Japanese cohort and also find a strong connection between *Lactobacillus iners* dominance and favorable reproductive results, while the presence of mixed flora is associated with a lower frequency of clinical pregnancy. In other research, however, such as this by Franasiak et al. (2016) [36], it is noted that not all patients with diverse microbiomes show negative reproductive results, which emphasizes heterogeneity in the data. Additional studies reveal geographical and ethnic variations. For example, Chen et al. (2017) [12] in the Chinese population discovered that in some women, *Lactobacillus crispatus* is dominant, while in others, *Streptococcus* or *Bifidobacterium* prevails; the association with fertility is not always unambiguous.

There is a lot of research emerging on the endometrial microbiome and its impact on receptivity. In 2021, Japanese scientists attempted to identify specific vaginal and endometrial microbiota in women with a history of recurrent implantation failure [11]. They concluded that disturbed microbiota communities containing specific bacteria in both the endometrium and vagina are associated with implantation failure. They suggested that the level of vaginal *Lactobacillus*, but not those in the endometrium, could be a biomarker for recurrent implantation failure (RIF). Two other research groups reported that the flora dominated by *Lactobacillus* appears to play a major role in determining fertility, and, in particular, *Lactobacillus crispatus* [32,37]. Despite the unclear causality, increasing evidence suggests that endometrial microbiota profiles may predict recurrent spontaneous abortion [38]. In a recent study, 67 women with two or more previous miscarriages underwent endometrial biopsies taken outside of pregnancy [39]. Analysis of microbiota profiles has shown that subsequent miscarriage is associated with increased relative dominance of *Ureaplasma*. A similar predictive effect of endometrial microbiota was previously described in a study of 342 women with infertility, which found that endometrial microbiota with predominant lactobacilli is associated with an increased likelihood of live birth [40,41]. Parvanov et al. (2023) investigated this relationship between the endometrial microbiome and the success of the implantation in 30 women after frozen embryo transfer [41]. They compare the endometrial microbiome between women who had become pregnant and non-pregnant women, as embryo transfer with euploid embryos was performed up to 3 months after endometrial biopsy. The authors found that the genus *Lactobacillus* does not significantly distinguish between the two studied groups, but specific bacterial taxa (*Serratia marcescens*, *Staphylococcus* spp., *Glutamicibacter* spp., and *Delftia* spp.) have a significantly higher relative abundance in the endometrium of patients with implantation failure. Other authors discuss emerging evidence supporting the theory that dysbiotic endometrial microbiota can indeed modulate key inflammatory pathways needed for successful implantation of the embryo and development of pregnancy [42]. Bellver et al. (2024) [43] studied the microbiota of the digestive and reproductive tracts in infertile women with obesity and found that vaginal and endometrial samples had a similar microbiota with the presence of *Lactobacillus*, *Gardnerella*, and *Streptococcus*, but in obese patients a higher frequency of *Streptococcus* (>50%) was observed in the endometrial microbiota, which may partly explain their poor reproductive outcomes.

A general trend emerging from multiple 16S-based studies is the distinction between a “*Lactobacillus*-dominant” and “non-dominant” microbiome profile, which is considered a potential biomarker for reproductive outcome. However, significant discrepancies between studies indicate that NGS-based 16S sequencing alone is not sufficient to fully understand the role of the endometrial microbiome.

During the last decade, **shotgun metagenomics** has been confirmed as a powerful tool for more deeply researching the endometrial microbiome, building on the restrictions of 16S rRNA sequencing [6,12]. For differences from amplicon approaches, shotgun analysis allows simultaneous taxonomic and functional characterization of microbial communities and reveals greater detail on the level of species and strains [14]. The first metagenomic studies show that although *Lactobacillus* spp. are often dominant, the endometrial microbiome also contains a number of low-abundant taxa that are not detected with 16S approaches [12].

Several independent studies demonstrate that in women with reproductive problems, specific microbial signatures, including anaerobes such as *Gardnerella vaginalis*, *Atopobium vaginae*, *Prevotella* spp., and *Streptococcus*, are discovered [40,44]. Shotgun analysis also tracks metabolic potentials of these bacteria, such as, for example, production of biofilm-associated enzymes, lipopolysaccharides, and other factors that could influence endometrial receptivity [45]. At the same time, studies of women with successful implantation and normal reproductive status show a stable presence of *Lactobacillus crispatus* and *L. gasseri*, as well as lower microbial diversity [13]. Table 3 shows the microbial profile of the endometrium in health and pathology.

Shotgun metagenomics detects both viral and fungal components in the endometrial environment, which usually remain invisible in 16S analyses [14]. Several reports show that herpes viruses and HPV may be found in uterine samples, which raises questions about their role in endometrial homeostasis [14]. Also, metagenomics data reveal potential functional pathways related to inflammation, immunomodulation, and hormonal regulation. Through a metatranscriptomic approach, Sola-Leyva et al. (2021) map the whole alive microbiota, consisting of is of >5300 microorganisms in the endometrium of healthy women [46]. There were significant differences in microbial content in the middle secretory compared to the proliferative phase of the endometrium.

The comparative analysis between different shotgun metagenome studies shows significant heterogeneity. For example, while some authors describe exceptional domination of *Lactobacillus* in the endometrium [6], others establish a richer microbiome with participation of *Bacteroides*, *Clostridium*, and even *Escherichia coli* [12,14]. These differences are probably owed to geographical and population features, as well as differences in protocols for sampling and bioinformatics analysis [47].

One of the key advantages of shotgun metagenomics is the ability to distinguish viable from nonviable bacteria by functional genes, although this is not yet routinely applied. In addition, metagenomics provides information on antibiotic resistance genes in the endometrial environment, which has direct clinical relevance in the treatment of chronic endometritis [48].

The general consensus among most shotgun studies is that the endometrium represents a low biomass, but biologically active environment in which exists dynamic interaction between bacteria, viruses, and host [14,47]. The evidence indicates that *Lactobacillus*, the dominant microbiome, is beneficial for implantation, while dysbiotic profiles with participation of anaerobes and conditionally pathogenic bacteria are associated with adverse reproductive outcomes [6,13].

Despite this, there remains a significant need for standardization in the analysis and interpretation of shotgun metagenomics data [31,49]. The lack of unified protocols makes it difficult to make direct comparisons between studies and places into question the reproducibility of results [47]. Future research must include bigger cohorts, control of contamination, and integration of metagenomic with metatranscriptomic and metabolomic data.

**Figure 1:**
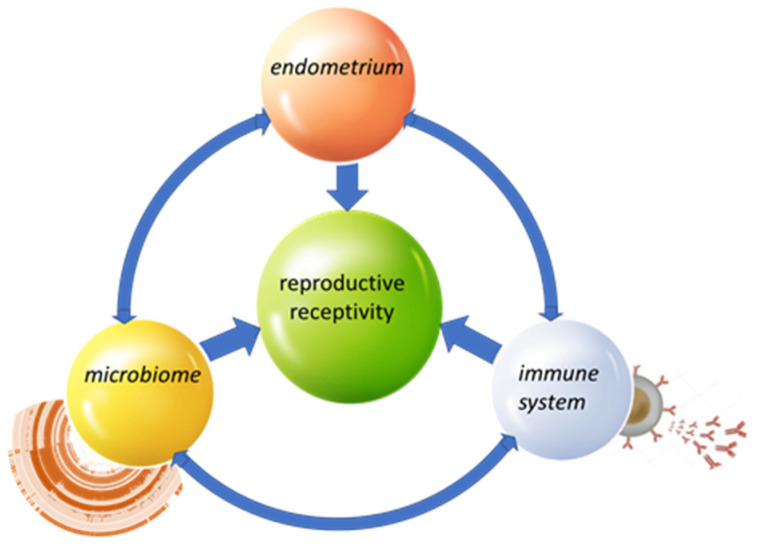
Key interactions.

In conclusion, shotgun metagenomics offers a unique perspective for understanding the endometrial microbiome, providing not only taxonomic but also functional information. It reveals a more complex and diverse ecosystem than previously thought, and suggests new avenues for clinical intervention, especially in the field of reproductive medicine. In the future, integration with metatranscriptomics and functional analyses will be required to gain a clearer picture of microbial–host interactions (Figure 1).

**Despite the differences in the studies published to date, several main conclusions can be drawn:** (1) The endometrium is not a sterile environment. The uterine cavity contains its own microbiome, albeit with a low biomass. (2) *Lactobacillus* spp. are dominant under physiological conditions. In most cohorts, a trend towards a predominance of *Lactobacillus* has been observed, which is associated with the maintenance of homeostasis and favorable reproductive potential. (3) Dysbiosis is associated with reproductive disorders. Increased microbial diversity and the presence of anaerobic bacteria such as *Gardnerella, Atopobium,* and *Prevotella* are often associated with chronic endometritis, implantation failure, and adverse IVF outcomes. (4) There are significant individual and population differences. Geographic, ethnic, and hormonal factors, as well as antibiotic exposure, may influence the composition of the endometrial microbiome, which explains some of the contradictory results.

## 4. Study of the Endometrial Microbiome Using Cultivation Methods: Isolation of Pure Cultures of “Beneficial” Microorganisms

Culturomics techniques allow the isolation and identification of viable microorganisms and provide mainly qualitative information on the species identity of the isolated microorganisms, while sequencing methods provide a quantitative profile of the microbiota. The limitations of culture techniques are related to the inability to cultivate some anaerobic or difficult-to-grow species in laboratory conditions [50]. On the other hand, only culture techniques allow the isolation, study, and storage of pure cultures of “good” microorganisms that could be used as probiotic strains and in biotherapy.

To date, culture techniques have been applied in a limited number of studies on endometrial samples, most of which focus on the identification of opportunistic pathogens, such as *Escherichia coli*, *Gardnerella vaginalis*, and *Streptococcus agalactiae* [29,50]. Demonstration of the microbiota in poorly studied anatomical areas remains a challenge.

In a 2025 study involving 50 women, 10 of whom were healthy as a control group and the rest had documented reproductive problems, microbiological analysis was performed by culturing samples from the endometrium, cervical canal, and vagina. In the control group, *Lactobacillus* representatives were isolated in 7.5% of the endometrial samples, while in women with fertility disorders, the frequency of *Lactobacillus* isolation was higher, 30% in the endometrium, respectively [51]. The authors compared the culture method with the qPCR method for the identified lactobacilli. The qPCR method showed a much higher positivity, with the frequency of identified lactobacilli in endometrial samples by quantitative real-time PCR in these two groups being 40%.

Cariati’s group [50] (2023) investigated the endometrial microbiota profile in 93 in vitro fertilization (IVF) patients using culturomics-based analysis and MALDI-TOF MS identification. In 74% of the patients, the presence of one or more microorganisms was detected, while in 26% no microbial growth was observed. They found that the phylum Firmicutes was the most frequently isolated taxon, and *Lactobacillus* spp. significantly correlated with ongoing pregnancy, while the presence of *Staphylococcus* spp. and *Enterobacteriaceae* had a negative impact on implantation rates. The following species were identified: *L. gasseri*, *L. crispatus*, *L. jensenii*, *L. iners*, *L. rhamnosus*, *L. fermentum*, *L. paracasei*, and *L. johnsonii* [50]. The bacterial population was assessed mainly by growth on solid media, with enrichment broths being used only in the absence of growth on agar media.

In a pilot study [52], the effectiveness of culturomics—a high-throughput cultivation approach—in the analysis of the endometrial microbiome was demonstrated. Ten women with reduced fertility who underwent diagnostic hysteroscopy and endometrial biopsy were included. Despite the low microbial density of the endometrial tissue, 83 bacterial and 2 fungal species were isolated and identified through culturomics, supported by the WASPLab^®^ system. MALDI-TOF mass spectrometry or 16S rRNA sequencing was used to identify the isolates. Two approaches were used to process the samples: one part was directly inoculated onto multiple aerobic and anaerobic media, while the second was subjected to pre-enrichment with two different incubation conditions (aerobic and anaerobic). Most of the isolated species (91%) matched genera already described in previous metagenomic studies of the endometrium. Representatives of the genus *Lactobacillus* were isolated from all 10 samples, including *L*. *coleohominis*, *L. crispatus*, *L. delbrueckii*, *L. gasseri*, *L. jensenii*, *L. iners*, and *L. vaginalis. L. jensenii* was the most frequently isolated species, confirming data from previous metagenomic analyses of the endometrial microbiome [52]. Culturomics provides an opportunity for deeper species differentiation—53 of the total isolated species (62.4%) were described for the first time as part of the endometrial microbiota. In the methodology applied for the isolation and identification of microorganisms by cultivation, the application of different incubation conditions is important: aerobic, anaerobic, or the need for pre-incubation of the samples in broth [33].

The same research team [53] conducted a further study comparing the endometrial and vaginal microbiomes by applying culturomics on paired samples using the WASPLab-assisted culturomics protocol. Ten women with reduced fertility who underwent hysteroscopy and biopsy were included. On average, 28% of the species were detected in both the endometrial biopsy and vaginal swab of the respective patient. Thus, a culturomics-based approach sheds further light on the current understanding of the endometrial microbiome, and the reported data suggest the potential existence of a unique endometrial microbiome that is not a consequence of cross-contamination obtained during sampling. All vaginal and endometrial samples contained one or more members of the genera *Lactobacillus*, *Ligilactobacillus*, or *Limosilactobacillus*. Among the species identified are *L. crispatus*, *L. gasseri*, *L. iners*, *L. jensenii*, *L. vaginalis*, *Lancefieldella rimae*, and *Limosilactobacillus fermentum. L. jensenii* is predominantly observed in microbiomes with low microbial diversity, while *L. iners* is more characteristic of microbiomes with high diversity, often associated with dysbiosis. The present data highlight the importance of optimized cultivation conditions and careful selection of the culturomics approach when analyzing the microbiota in sparsely populated microenvironments such as the endometrium [53].

*Lactobacillus* spp. are the dominant colonizers in healthy women of reproductive age, maintaining an acidic pH (below 4.5) and limiting the spread of pathogens through the production of organic acids, hydrogen peroxide (H_2_O_2_), antimicrobial compounds (bacteriocins), biosurfactants, exopolysaccharides, and disruption of biofilms [54,55,56,57,58].

Although the protective function of *Lactobacillus* in the vaginal ecosystem is well documented, the mechanisms by which this genus affects fertility remain incompletely understood and are the subject of active investigation [33]. *Lactobacillus*-associated metabolites have demonstrated antagonistic activity against a wide range of bacterial and fungal pathogens, including *Candida albicans*, *Staphylococcus aureus*, *Streptococcus mutans*, *Escherichia coli*, *Pseudomonas aeruginosa*, and *Salmonella typhimurium* [59]. Based on the accumulated data, the selection and application of *Lactobacillus* strains with optimal characteristics—including the ability to rapidly acidify the environment, effective pathogen suppression, and high adhesive activity to the mucosa—may represent an effective approach to restore microbial balance in the female reproductive tract.

The use of vaginal probiotics composed of well-selected strains isolated from the genital tract may not only help restore a healthy microbiota, but also have a positive impact on the upper parts of the female reproductive system [60]. The reason for this is that standard probiotics are usually not adapted to the specific conditions of the genital tract and fail to sustainably normalize the environment. As a result, the microbiome remains unstable, and dysbiosis-related conditions occur recurrently.

Some authors suggest a link between the intestinal microbiome and the microbiome of the reproductive tract [61]. Sobstyl et al. [62] (2022) commented on data suggesting that gynecological and gastrointestinal dysbiosis may play an active role in the development and metastasis of gynecological neoplasms, such as cervical, endometrial, and ovarian cancer. Baușic et al. [63] (2025) found dysbiosis of the gut microbiota in endometriosis with increased levels of Bacteroidetes and increased inflammatory markers, such as β-glucuronidase and secretory IgA, and suggested a potential link between the gut microbiota and systemic inflammation in endometriosis. Barczyński et al. [64] (2023) investigated the composition of the vaginal and cervical microbiota in patients with endometrial cancer, and their data indicated that *Lactobacillus iners* was significantly more common in patients with benign disease, while *Dialister pneumosintes* and *Mobiluncus curtisii* were more common in patients with cancer. According to the authors, understanding the role of the microbiome in endometriosis could open new avenues for noninvasive diagnostic tools and microbiota-targeted therapies. Advances in understanding the human microbiota have significantly accelerated the development of innovative approaches in live biotherapy, including the use of probiotics and entire communities of microorganisms with therapeutic potential [60,65,66].

## 5. Therapeutic and Clinical Applications

Disruption of the dominance of *Lactobacillus* spp. in the vaginal microbiome is associated with increased bacterial diversity and, consequently, a higher risk of gynecological problems, including bacterial vaginosis, infections, and adverse pregnancy outcomes (premature birth, miscarriage) [67].

A probiotic with *L. crispatus* has been shown to significantly improve vaginal health in women with bacterial vaginosis [68]. This study noted that after treatment, dysbiosis was reduced and the balance of the vaginal microbiota improved, suggesting the possibility of restoring the dominant *Lactobacillus* flora [68]. Another study evaluated the safety of a vaginal probiotic (*L. crispatus* CTV-05) in pregnant women at high risk of preterm birth [69]. It was reported that probiotic administration was well tolerated and no serious adverse events were observed, supporting the potential for interventions targeting microbiome therapy to reduce risks during pregnancy [69]. A study by Kadogami, 2020, examined the effect of vaginal probiotics in combination with antibiotic therapy in women with endometrial microbiota disorders (non-*Lactobacillus* dominated profile) [70]. The authors found that combining vaginal probiotic suppositories (inVag: *L. gasseri*, *L. fermentum*, and *L. plantarum*) with an antibiotic was more effective than single-agent therapy in restoring the endometrial microenvironment. In a 2024 study, Sakamoto et al. [71] reported the effectiveness of combining antibiotics and probiotics *(L. acidophilus* La-14 and *L. rhamnosus* HN001 strains) in vaginal formulations for the treatment of abnormal uterine microflora. A recent publication [72] describes the study of a strain of *L. crispatus* M247 isolated from a probiotic product, which showed strong antimicrobial properties against various pathogens. Its cell-free supernatant (CFS) was found to be more effective than the live strain itself. In in vitro experiments, CFS significantly reduced the growth of pathogens such as *Escherichia coli*, *Klebsiella pneumoniae*, *Staphylococcus aureus*, *Streptococcus agalactiae*, *Enterococcus faecalis*, and *Candida albicans*. This suggests that CFS therapies may be more successful than the oral administration of live bacteria. A study by Vander Donck et al. (2025) showed that different *Lactobacillus* spp. can stably coexist through metabolic interactions, which provides a basis for future microbiome therapies [73].

Probiotics containing *Lactobacillus* strains exert their beneficial effects on the host through a complex network of microbiological, immunological, and metabolic mechanisms that collectively support mucosal homeostasis and reproductive health. These bacteria act primarily by competitively excluding pathogenic microorganisms, occupying epithelial adhesion sites, competing for nutrients, and forming biofilms that create a physical and biochemical barrier against harmful species such as *Gardnerella*, *Escherichia coli*, and *Candida*. In addition, *Lactobacillus* strains produce antimicrobial substances including lactic acid, which lowers the local pH and inhibits the growth of pathogens; hydrogen peroxide (H_2_O_2_), which disrupts microbial membranes through oxidative stress; and bacteriocins—small peptides that selectively suppress competing bacteria [54,56,57]. These probiotics also modulate the host immune system by promoting anti-inflammatory cytokine responses (e.g., IL-10), downregulating pro-inflammatory mediators such as IL-6 and TNF-α, stimulating mucosal immunity through enhanced IgA secretion, and interacting with toll-like receptors to support immune tolerance [55,57]. Furthermore, *Lactobacillus* species strengthen the epithelial barrier by upregulating tight junction proteins, stimulating mucin secretion, and reducing epithelial permeability, thereby preventing pathogen translocation [58,59]. Their metabolic activity contributes to host health through the production of short-chain fatty acids like acetate and butyrate, which possess anti-inflammatory and tissue-repairing properties, as well as by modulating oxidative stress and influencing estrogen metabolism within reproductive tissues. Additionally, *Lactobacillus* strains engage in cross-talk with other beneficial microorganisms such as *Bifidobacterium*, fostering a balanced and resilient microbial community that supports vaginal and endometrial homeostasis [56,58]. Altogether, *Lactobacillus* probiotics are key modulators of the reproductive tract environment, promoting microbial balance, epithelial integrity, and immune regulation.

Although many authors suggest that increasing the level of *Lactobacillus* occupancy in the uterus leads to improved IVF outcomes, there are no published studies that have directly investigated this relationship. Furthermore, there are no studies comparing the effectiveness of different *Lactobacillus* spp. contained in probiotics. Considering that the endometrial microbiome can originate from other parts of the body (vaginal, oral, intestinal, etc.), from a general perspective, probiotic treatment in other parts of the body may also be useful for regulating the balance of the endometrial microbiome. There is evidence that the use of probiotics affects various biological processes associated with chronic endometritis [74] and tumorigenesis in endometrial cancer (inflammation, oxidative stress, apoptosis, proliferation, and metastasis) [75].

Based on these studies, several key conclusions can be drawn: first, dysbiosis—defined as a decline in *Lactobacillus*-dominated microbiota and an enrichment of anaerobes and opportunistic pathogens—correlates with adverse gynecological and reproductive outcomes. Second, *Lactobacillus crispatus*-based interventions show promising results in terms of symptom reduction and improvement of the microbiome ecosystem. Third, probiotic approaches, according to available data, are safe in specific groups (pregnant women at risk), which is critical for future clinical applications.

However, these studies focused primarily on the vaginal microbiome and vaginal infections, and not explicitly on the endometrial flora and its relationship to reproductive outcomes such as implantation success or live birth. Also, although a reduction in dysbiosis and an enrichment of the vaginal flora have been shown, it is unclear to what extent these effects translate to uterine conditions or directly affect endometrial receptivity.

In conclusion, the body of evidence supports the idea that restoring and maintaining a *Lactobacillus*-dominant microbiota is a promising strategy for reducing gynecological and reproductive risks. However, gaps remain—especially with regard to direct evidence for the endometrium—requiring future studies with specific endometrial samples and functional assays to form the basis for personalized reproductive medicine based on a microbiome profile.

## 6. Immunological Mechanisms—Research Methods: Interaction of the Microbiome with the Immune System and the Local Environment

The implantation of the human embryo represents an extremely complex and strictly regulated biological process that needs synchronized interaction between the embryo and the endometrial mucous membrane. The successful implantation is happening in a strictly limited period known as the “implantation window”, during which the endometrium becomes receptive to the blastocyst. The disturbances in this fine balance would lead to repeated implantation failures (RIF), early abortions, or infertility. The endometrial receptivity and the early implantation are also dependent on the communication with the epithelial cells, immune cells, and the microbiome. Because of the ethical and practical limitations, it is impossible to study in vivo in women the basic aspects of the endometrium differentiation and regeneration, and the pathological aspects of the implantation also. The use of different animal models could provide some important insights, but they could not completely reproduce the biology of the human endometrium. To fill the significant hole in understanding these complex communications, the in vitro models of endometrium were established.

The human endometrium contains two different cell types: stromal and epithelial cells. The inner surface of the uterine epithelium is differentiated as luminal epithelium (LE). The first contact of the embryo trophectoderm is with the LE. The invaginations of the epithelium form glands that form glandular epithelia (GE) that produce factors important for the embryo’s survival, the growth of the placenta, and also have an impact on stromal decidualization [76].

### 6.1. Human Endometrial Epithelial Cell Lines

Since the last decades, the in vitro models of endometrial cell lines like Ishikawa, RL95-2, and HEC-1A have become approved instruments for studying the endometrial receptivity, the blastocyst’s adhesion, and the molecular mechanisms that regulate this process. They are widely used because of their acceptability, stability in culture, and reproducibility of the results, although there are some restrictions according to their tumor origin. During the menstrual cycle, the human endometrium changes its polarity, adhesion, and cytoskeletal organization [77]. Depending on these characteristics, endometrial cell lines with different degrees of polarization were used. The HEC-1 cell line is an example of a non-receptive cell with low adherent properties and high polarization properties [77,78]. A cell line with opposite characteristics, like high receptivity and high adhesive properties, is the RL95-2 epithelial cell line [77,78].

Another well-differentiated human endothelial adenocarcinoma cell line, established more than 20 years ago and widely used as a good model for studying normal endothelial function, is the Ishikawa cell line. This cell line possesses mixed characteristics of LE and GE. With its adhesiveness, moderate polarization, and the expression of functional steroid receptors, the Ishikawa cell line is one of the most used ones [77,78,79]. According to its LE properties, the Ishikawa cell line expresses MUC1 cytokeratins (KRTs) 13 and 18, which makes it a good model for luminal epithelium.

Two other cell lines with LE characteristics are the ECC-1 cell line and the HES line. They also express cytokeratins (KRTs) 13 and 18, estrogen receptors alpha and beta, progesterone receptors A and B, and androgen receptors [78].

KLE is a cell line that was isolated from the endometrium of a 64-day-old female patient with undifferentiated endometrial adenocarcinoma. It is characterized by low receptivity and lower adhesiveness toward trophoblast cells, and that makes it more suitable for studying the molecular pathways of tumorigenesis and cell differentiation than as a model for implantation [80].

The AN3 CA cell line is a human endometrial epithelial cell line developed from poorly differentiated endometrial adenocarcinoma. This line demonstrates low adhesive properties and reduced receptivity, which determines it as a “nonreceptive model”. The AN3 CA line is used for cell signaling research, molecular regulators of cell growth and migration, as well as evaluating the effects of various cytokines and hormones on endometrial epithelial function [81].

These cell lines are usually cultivated under standard conditions—37 °C, 5% CO_2_, with culture media such as DMEM/F12 or McCoy’s, enriched with 10% FBS and antibiotics. The line RL95-2 requires the addition of insulin, which is important for maintaining its morphology and functional characteristics.

Although there are limitations according to the origin of these cell lines, they are valuable instruments for studying the cellular and molecular mechanisms, including the expression of adhesion molecules, cytokine secretion, and the interaction with the trophoblast cells. They also serve as a basis for the development of more complex models, such as co-culture systems with immune cells and 3D organoids, which more accurately reflect the complex physiology of the endometrium.

### 6.2. Three-Dimensional Human Endometrial Culture Models

Since the 1980s, the development of the 3D human endometrial culture models has led to significant progress in the research of the human endometrium’s receptivity. Unlike the traditional two-dimensional (2D) cell culture methods, which often lose tissue-specific specificities, 3D models allow the reconstruction of the architecture and functionality of the endothelium. This makes them particularly valuable for studying processes such as blastocyst implantation, the interaction between epithelial, stromal, and immune cells, and the role of the microbiota and inflammatory mediators.

Different types of 3D cultures could be distinguished depending on the organization, the matrix, and cell content: spheroids, organoids and co-culturing systems—assembloids.

**Spheroids** are 3D aggregates of endometrial cells (epithelial, stromal, or a mixture of them) that are self-organized while cultivated in suspension or on a matrix [82,83]. These organizations imitate the steric organization and cell–cell contacts in the tissue better than the 2D cultures. The spheroids are often used in co-culture with trophoblast cells or embryo spheroids to modulate the early stages of adhesion and invasion of the blastocyst [84]. These systems allow for the evaluation of the expression of the adhesion molecules like integrins, osteopontin, etc., and the role of cytokines and hormones on embryo-endometrium interactions [85].

**Organoids** are more complex 3D structures that contain primary endometrial epithelial stem cells or progenitor cells, cultivated in the presence of a specific matrix, like Matrigel, and in the presence of medium containing factors supporting cell differentiation [86]. The formed gland-like structures reproduce the cyclical changes in the endometrium under the influence of estrogen and progesterone [87]. The organoids preserved tissue-specific markers, the functional hormonal response, and the secretory activity, and all these features make these structures exceptionally valuable to studying the receptivity, the “implantation window”, and pathological conditions like endometriosis, chronic endometritis, or recurrent implantation failure (RIF) [88,89].

**Assembloids** are the next most complicated form of 3D models that combine endometrial organoids with stromal cells and/or immune cells in a common 3D structure. This complex culture model reproduces the cell diversity of the endometrium and its dynamics. Assembloids enable the study of interactions between epithelium, stroma, and immune cells, which is critical for successful implantation. They are used to study endometrial pathology, infertility, and to test targeted therapies [88,89].

The main differences between the 3D human endometrial models are summarized in Table 4.

Three-dimensional human endometrial cultures are a powerful tool for studying the mechanisms of implantation and endometrial pathologies. They provide a significantly closer to in vivo environment compared to classical cell lines, but their application still requires standardization and optimization. In the future, these models will likely be integrated with new technologies such as microfluidic systems (organ-on-a-chip) and multiomics analyses to achieve a better understanding and therapeutic application in reproductive medicine.

### 6.3. Interaction Between the Endometrial Microbiota and Endothelium

The endometrial endothelium functions as both a physical barrier and an active immunological sensor. Epithelial cells express pattern recognition receptors, including Toll-like receptors, which detect microbial components and trigger intracellular signaling cascades leading to the production of cytokines and chemokines such as IL-1b, IL-6, and TNF-a. The immune signaling shapes the local environment to either support or hinder blastocyst adhesion. A key condition for successful embryo implantation is synchronization between the stage of embryo development and the condition of the endometrium. Recently, more and more data have emerged on the importance of the endometrium microbiota for embryo development, its attachment/positioning/invasion, and early pregnancy. Some studies analyze whether the endometrial microbiota can influence the prenatal conditions in the mother and subsequently affect endometrial receptivity [90,91].

The female reproductive tract contains 9–10% of the entire human microbiome population. The diversity of the microbiome is greater from the vagina to the ovaries, with decreasing bacterial diversity from the external to the internal organs [92]. Microbiota diversity in the female genital tract changes throughout a woman’s life, influenced by factors such as age, ethnicity, sexual activity, and overall health status [20]. Hormonal fluctuations of progesterone and estrogen during the menstrual cycle also shape the endometrial microbiota. An increase in the microbial abundance has been observed during the proliferation phase, while the composition appears stable during the days critical for endometrial receptivity [25]. The glycogen deposition in the epithelium of the vagina is caused by estrogen, whereas progesterone-induced cytolysis facilitates its release into the lumen. The liberated glycogen is metabolized by *Lactobacillus* spp. into lactic acid, thereby maintaining an acidic vaginal environment (pH 3.5–4.5), which is critical for microbial homeostasis and protection against pathogens [93]. It’s already shown that the predominance of *Lactobacillus* spp. is associated with more favorable reproductive outcomes, while dysbiosis (increase in anaerobes such as *Gardnerella*, *Atopobium*, and *Prevotella*) is associated with chronic endometritis, reduced receptivity, and lower implantation rates [6,26]. Alterations in the microbiome may contribute to the pathogenesis of endometriosis by modifying the local microenvironment. As emphasized in the work of Sirota et al. [94], bacterial-driven uterine inflammation can disrupt the cytokine balance required for proper blastocyst development and successful implantation. In particular, dysbiosis has been associated with increased production of cytokines that promote inflammation (pro-inflammatory), such as IL-6 and TNF-α, which may impair endometrial receptivity, while downregulation of implantation-supporting mediators like leukemia inhibitory factor (LIF) further compromises the adhesion of the blastocyst to the endometrial epithelium [95].

The interaction between the endometrial microbiome and the endothelium occurs through a complex network of immunological, metabolic, and cell-molecular mechanisms that can either support or hinder successful implantation. One major pathway is **immunological modulation**. Endometrial epithelial cells express TLR2, TLR4, and other pattern recognition receptors that detect bacterial components such as LPS and peptidoglycans [96]. In a state of eubiosis, *Lactobacillus* spp. help maintain a local environment that suppresses the immune response against specific antigens (a tolerogenic environment) by stimulating the production of anti-inflammatory cytokines such as IL-10 and TGF-β. Dysbiosis, in contrast, promotes elevated levels of pro-inflammatory cytokines, including IL-1β, IL-6, and TNF-α, which may impair endometrial receptivity [97]. The microbiome also influences the activity of uterine natural killer cells (uNK) and M2 macrophages, which are crucial for angiogenesis and immune tolerance during implantation.

**Metabolic mechanisms** represent another key mode of interaction. *Lactobacillus* spp. metabolize glycogen into lactic acid, lowering vaginal and endometrial pH and creating a protective antimicrobial environment [90]. Short-chain fatty acids produced by certain bacterial species can regulate the epigenetic programming of endothelial cells and modulate the expression of adhesion molecules. Conversely, dysbiotic bacteria, such as *Streptococcus* and *Staphylococcus*, may release metabolites that induce oxidative stress or apoptosis in endometrial cells, disrupting tissue homeostasis. *Lactobacillus*-dominant microbiota enhances epithelial tight junction integrity, whereas dysbiosis may increase epithelial permeability. Additionally, microbial interactions stimulate the secretion of antimicrobial peptides such as defensins, contributing to local immune homeostasis.

Finally, **epigenetic and signaling regulation** also play an important role. Microbial metabolites and inflammatory mediators can influence DNA methylation and histone modifications in endothelial cells, altering the expression of genes related to receptivity, angiogenesis, and inflammatory responses. These changes highlight the intricate crosstalk between the microbiome and the endometrium, demonstrating how microbial composition can directly affect implantation outcomes.

### 6.4. Pro-Inflammatory Molecules, Cytokines, and Implantation Factors

The window of implantation that happens typically between days 20 and 24 of a normal menstrual cycle represents the period during which the endometrium becomes receptive to embryo implantation. During this short period, the luminal epithelium represents the first point of contact with the embryo and plays a decisive role in implantation success [24]. Over the past decade, the application of omics technologies has greatly advanced the understanding of endometrial receptivity. Genomic approaches using high-throughput sequencing have led to the identification of numerous receptivity-associated genes. The Human Gene Expression Endometrial Receptivity database (HGEx-ERdb) currently catalogs over 19,000 endometrial genes, of which 179 have been consistently associated with receptivity across multiple studies [98].

Epigenomics, transcriptomics, and proteomics have provided further insight into the dynamic regulation of endometrial receptivity during the menstrual cycle. Epigenetic modifications, such as DNA methylation and histone modifications, contribute to the cyclic expression of implantation-related genes. Transcriptomic profiling has identified gene expression signatures characteristic of the receptive phase, while proteomics studies reveal post-transcriptional and post-translational regulation of adhesion molecules, cytokines, and growth factors essential for blastocyst attachment. Twelve genes are potentially significant for endometrial receptivity, such as Calpastatin (*CAST*), Cystic Fibrosis Transmembrane Conductance Regulator (*CFTR*), Fibroblast Growth Factor Receptor 2 (*FGR2*), and *LIF* [99]. Recent studies have validated 19 miRNAs, such as members from the miR-30 and miR-200 families, and 11 of them were upregulated and involved in endometrial receptivity [100]. The human endothelial stromal cells are in extensive communication with the blastocyst and serve as biosensors of the embryo quality. They respond to the embryo-derived hsa-miR-320a by changing their migratory capacity. This function of the endothelial stromal cells serves as an implantation checkpoint. On the other hand, the communication of the endometrium epithelium activates the differentiation of the syncytiotrophoblast and expression of adhesion molecules. The disturbance of the communication between the endometrium and the embryos may also cause problems such as RIF.

Embryo implantation is a highly regulated process that requires a delicate balance between pro-inflammatory signaling, immune tolerance, and tissue remodeling. The endometrium undergoes a transient, inflammatory-like response during the implantation window, with cytokines and growth factors orchestrating successful blastocyst attachment and invasion. Among the key cytokines, IL-1 (α/β) plays an essential role by promoting adhesion molecule expression on endometrial epithelial cells and facilitating extracellular matrix remodeling [101,102]. IL-6 contributes to decidualization, angiogenesis, and trophoblast invasion [101,103], while TNF-α exhibits dual effects: at physiological levels, it supports implantation, but excessive levels can impair trophoblast survival and lead to pregnancy complications [101,104]. Another critical cytokine is LIF (Leukemia Inhibitory Factor), indispensable for endometrial receptivity and blastocyst adhesion [101,102,104]. IFN-γ, largely produced by uterine natural killer (uNK) cells, modulates immune responses and participates in vascular remodeling [105,106]. Similarly, chemokines such as MCP-1 (CCL2) help recruit immune cells to the implantation site, reinforcing the localized inflammatory environment [101].

In addition to cytokines, a broad range of implantation factors contribute to the establishment of receptivity. Integrins (e.g., αvβ3, α4β1) mediate firm blastocyst adhesion to the endometrial epithelium [102,104], while MUC1, a large glycoprotein, is downregulated locally to permit attachment [103,104]. VEGF ensures proper angiogenesis and vascular permeability, supporting early placental development [102,103]. TGF-β regulates immune tolerance and extracellular matrix remodeling [101,104], whereas members of the EGF family stimulate trophoblast proliferation and invasion [101,102]. Transcription factors NOXA10 and NOXA11 further regulate endometrial receptivity by influencing stromal differentiation [103,104]. Enzymes like COX-2 and prostaglandins (PGE2, PGF2a) mediate vascular permeability and decidualization, facilitating embryo implantation [102,106]. Finally, immune mediators such as uNK cells and GM-CSF are central in promoting immune tolerance, vascular remodeling, and trophoblast growth [103,105,106].

Overall, implantation depends on the precise temporal and spatial regulation of these molecules. Insufficient inflammatory signaling can result in poor receptivity and implantation failure, whereas excessive inflammation is linked to recurrent pregnancy loss and pathologies such as endometriosis-related infertility [101,102,106].

### 6.5. Immunological Markers That Determine the Decidual Immune Profile and the Success of Implantation

The maternal–fetal interface represents a unique immunological environment in which tolerance and defense must be balanced. The decidua contains specialized immune cell populations—primary uterine natural killer (uNK) cells, macrophages, T cells, and dendritic cells—that shape the immune profile required for successful implantation and early placentation [101,106]. The uterine natural killer cells are the most abundant leucocytes in early pregnancy and secrete cytokines such as IFN-γ, VEGF, and angiopoietins, which are critical for spiral artery remodeling and vascular adaptation to support the developing embryo [106,107]. Their activity is modulated by KIR (Killer Cell Immunoglobulin-like Receptors) on maternal uNK cells interacting with HLA-C, HLA-E, and HLA-G molecules expressed by trophoblasts, a process critical for immune tolerance and vascular regulation [107,108]. Dysregulations of this interaction have been linked to recurrent implantation failure and preeclampsia [108].

Macrophages contribute to implantation success by shifting toward an M2 (anti-inflammatory/tolerogenic) phenotype, characterized by the expression of IL-10, TGF-β, and CCL18, which promote tissue remodeling, angiogenesis, and immune tolerance [101,109]. Conversely, excessive M1-like activity, associated with TNF-α and IL-12 production, may impair trophoblast invasion and contribute to pregnancy complications [109].

Regulatory T-cells, defined by CD4+CD25+FoxP3+ expression, expand during the implantation window and secrete IL-10 and TGF-β, both of which are essential for maintaining tolerance to paternal antigens [106,110]. A reduction in frequency of the regulatory T-cells correlates with implantation failure and recurrent pregnancy loss [110].

Another important marker is the expression of HLA-G, a non-classical MHC class I molecule expressed by extravillous trophoblasts. HLA-G interacts with immune cell receptors such as ILT2 and KIR2DL4, suppressing NK and T-cell cytotoxicity, thereby contributing to maternal tolerance [108,110].

Taken together, the decidual immune profile—shaped by the balance between uNK cells, macrophages, Tregs, and trophoblast HLA molecules—acts as a determinant of implantation success. Disruption in any of these immunological markers can shift the environment toward excessive inflammation or inadequate tolerance, leading to impaired implantation outcomes [106,107,108,109,110].

## 7. Oxidative Stress and the Female Reproductive System

Notwithstanding considerable progress in assisted reproductive technologies (ART), their success rates continue to fall short of expectations. One of the major factors affecting the outcomes of antiretroviral therapy (ART) is oxidative stress (OS) [111].

### 7.1. The Nature of Oxidative Stress

Oxidative stress is defined as an imbalance between the production of reactive oxygen species (ROS) and antioxidant defenses [112]. The major ROS include superoxide anion (O_2_^•^), hydroxyl radical (^•^OH), and hydrogen peroxide (H_2_O_2_). Murphy (2009) [113] found that approximately 2% of the oxygen utilized in mitochondrial energy metabolism is converted to ROS. Increased levels of these molecules have been observed in a number of diseases and under the influence of harmful factors such as smoking, obesity, and unbalanced nutrition [114,115,116]. The present study investigates the physiological and pathological role of ROS in the field of reproduction. Reactive oxygen species (ROS) can induce oxidative damage to cells, leading to disruption of cellular membranes, accelerated aging, immune dysfunction, and alterations in intracellular signaling pathways [117].

Concurrently, controlled levels of ROS are implicated in the normal physiology of the female reproductive system, including folliculogenesis, oocyte maturation, ovulation, steroidogenesis, endometrial changes, and fetoplacental development [118]. However, excessive formation of ROS has been demonstrated to result in oocyte apoptosis, DNA damage, impaired implantation, and even pregnancy loss [119]. Postovulatory oocytes exhibit heightened sensitivity to the effects of ROS. The accrual of oxidative stress has been demonstrated to expedite their senescence, a process typified by alterations in the zona pellucida and the activation of ovoperoxidase [118,120].

With age, oxidative stress progressively increases, correlating with a higher incidence of aneuploidy due to chromosomal abnormalities in aging oocytes [118]. Accumulated damage to DNA, lipids, and proteins impairs cellular function and reproductive capacity [121].

The presence of oxidative stress in follicular fluid and endometrial secretions affects oocyte quality, embryonic development, and embryo transfer success [122,123,124]. Membrane fluidity plays an essential role in the regulation of cellular functions such as transport, enzymatic activity, signal transduction, and cell cycle [125]. At low concentrations, ROS function as secondary mediators in redox-dependent signaling cascades. However, when accumulated, they activate pathways such as mitogen-activated protein kinase (MAPK), which can initiate premature cellular senescence [126]. OS markers are used to predict the outcome of in vitro procedures, with higher antioxidant capacity being associated with an increased chance of clinical pregnancy [127].

Oxidative stress (OS) has a detrimental impact on several key aspects of female reproductive function:

- Oocyte quality: OS induces apoptosis, mitochondrial dysfunction, and chromosomal anomalies, compromising oocyte viability and developmental potential.
- Fertility: It contributes to chromosomal non-disjunction and increases the risk of aneuploidy, particularly in advanced maternal age [128].
- Steroidogenesis: OS suppresses the activity of granulosa cells and impairs the synthesis of essential reproductive hormones such as follicle-stimulating hormone (FSH) and estradiol [118].

Moreover, external factors such as psychological stress and aging exacerbate oxidative imbalance, further impairing fertility outcomes [118].

### 7.2. Oxidative Stress and Cellular Aging in Endometriosis

The role of ROS in endometriosis, a chronic inflammatory condition affecting 10–15% of women of reproductive age and up to 90% of infertile patients, deserves special attention [129,130,131]. The inflammatory processes that are characteristic of the disease have been shown to stimulate the overproduction of ROS, which in turn leads to damage to cell membranes, immune dysfunction, and impaired implantation [116,132].

Accumulation of ROS and activation of MAPK signaling pathways (ERK1/2, JNK, p38) have been proven in endometriosis, leading to cellular aging and metabolic dysfunction [133,134,135]. Elevated levels of ROS markers have been detected in endometrial, peritoneal, and follicular fluids, as well as in reproductive tissues.

Endometriotic lesions show persistent redox imbalance throughout the menstrual cycle, as well as increased expression of aging markers. Mechanisms include lipid and protein damage, changes in cellular fluidity, and signal transduction associated with aging and inflammation [117].

The relationship between oxidative stress and the female reproductive system is presented in Table 5.

### 7.3. MAPK Signaling and Aging

The process of ROS-induced growth arrest and the subsequent development of a secretory phenotype associated with senescence (SASP) is facilitated by the activation of the ERK1/2, JNK, and p38 cascades [141]. Increased activity of these pathways, especially ERK1/2, has been reported in endometriosis at different phases of the menstrual cycle [134,135]. This assertion is corroborated by in vitro experiments with stromal cells and GWAS analyses, which identify MAPK and, in particular, ERK1/2, as a pivotal factor in the pathogenesis process [142].

As demonstrated in the studies conducted by Borodkina et al. (2014, 2016), sublethal oxidative stress has been shown to induce premature senescence in endometrial stem cells by activating the p38 pathway [143,144]. The partial restoration of proliferation subsequent to p38 inhibition serves to substantiate the significance of this particular mechanism.

**Figure 2:**
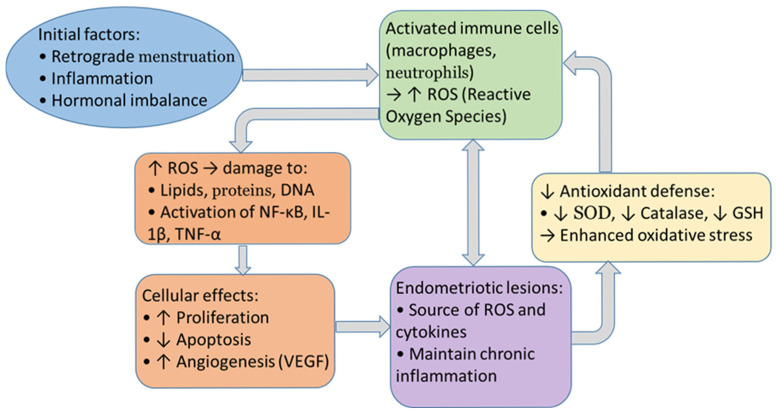
Cycle between oxidative stress and endometriosis.

This study investigates the relationship between ROS, MAPK, and endometriotic lesions (Figure 2). The presence of lesions in cases of endometriosis has been shown to be indicative of increased levels of reactive oxygen species (ROS), activated mitogen-activated protein kinase (MAPK) signaling, and elevated levels of markers of cellular senescence. Furthermore, stromal cells from such lesions exhibit an enhanced response to H_2_O_2_ compared to controls, thus emphasizing the role of OS in pathogenesis [144,145].

According to Malvezzi et al. (2023) [117], H_2_O_2_ stimulation activates MAPK pathways and leads to senescence in endometriotic stromal cells. Concurrently, eutopic endometrium from patients diagnosed with endometriosis exhibited diminished levels of ROS and MAPK, accompanied by an augmented prevalence of double-stranded DNA breaks (DSBs) [146].

### 7.4. Decidualization and Fertility

Deryabin (2021) [147] and Lessey (2017) [148] posit that impaired decidualization in endometriosis, possibly associated with stromal cell senescence, is a factor in reduced fertility. The increased presence of natural killer (NK) cells in the eutopic endometrium of patients with endometriosis indicates chronic stress or tissue damage [149]. Redox homeostasis in the endometrium is imperative. A slight increase in lipid peroxidation has been observed in the premenstrual phase [59,60], with the controlled presence of ROS being necessary to maintain cellular homeostasis.

It is imperative to acknowledge the pivotal role of the cellular antioxidant defense system in preserving cellular homeostasis.

### 7.5. Antioxidant Defense

The antioxidant system includes both enzymatic (SOD, CAT, GPx) and non-enzymatic components (e.g., glutathione). SOD catalyzes the conversion of superoxide to hydrogen peroxide, while CAT and GPx neutralize it to water and oxygen [136,150].

Higher levels of antioxidant enzymes (e.g., SOD, CAT, TAP) and lower levels of oxidative stress markers (e.g., LPO) have been found to correlate with better IVF outcomes [116]. Furthermore, the role of antioxidant defense in maintaining endometrial receptivity has been demonstrated [151,152].

### 7.6. Antioxidant Activity of Lactobacilli

Both whole cells and cell lysates of various *Lactobacillus* spp. have been shown to possess antioxidant properties; however, significant variation in the individual properties of DPPH, TAALA, SOD, and GSH is observed within and among different *Lactobacillus* strains. In general, all *Lactobacillus* strains tested, with the exception of *L. oris* and *L. gasseri*, exhibited relatively high scores across all antioxidant parameters, suggesting that antioxidant properties are strain-specific. However, it has been demonstrated that only certain *Lactobacillus* strains possess the capacity to manifest antioxidant activity [153,154,155,156].

A number of studies have reported that certain *Lactobacillus* strains can be a good natural source of antioxidants, and some strains have also been proven in in vivo studies [157,158]. Probiotic supplementation in rats has been demonstrated to induce the transcription of genes involved in GSH biosynthesis in the intestinal mucosa [159] and to increase GSH synthesis in pancreatic cells [160]. As indicated in the seminal study by Peran et al. (2007) [161], comparable efficacy in restoring GSH levels after oxidative stress has been previously reported in rats supplemented with probiotic strains. *Lactobacillus fermentum* ME-3 has been demonstrated to elevate the redox ratio of GSH in blood serum [162], while *Lactobacillus casei* has been shown to curtail lipid peroxidation, thereby enhancing blood lipid metabolism [163].

Certain strains of *Lactobacillus* have been shown to exhibit elevated antioxidant parameters (Table 6).

## 8. Conclusions

From the scientific research reviewed on the topic, the following main points and guidelines can be summarized:

- Restoration and maintenance of *Lactobacillus*-dominant microbiota is a promising strategy for reducing gynecological and reproductive risks;
- Probiotic approaches show good results in terms of reducing symptoms and improving the microbiome ecosystem of the reproductive tract, but so far, these studies have focused primarily on the vaginal microbiome and vaginal infections, and not explicitly on the endometrial flora and its relationship with reproductive outcomes;
- The endometrial microbiome may influence the local immune response in the uterus, which is important for protection against pathogens and for tolerating pregnancy;
- Standardization of studies is needed to avoid methodological problems and distortion of results;
- One of the main factors influencing reproductive susceptibility is oxidative stress;
- Some strains of *Lactobacillus* exhibit relatively high antioxidant parameters, thereby favoring stress management and improving reproductive capacity.

## Figures and Tables

**Table 1:** Chronology from “sterile uterus” to a new paradigm.

Year	Authors/Research	Main Contribution
<2000	Numerous studies using culture techniques (samples from uterus, placenta, cesarean sections)—Paavonen et al. 1986 *AJOG*, Møller et al. 1995 *AOGS*, Bearfield et al. 2002 *BJOG*, Stout et al. 2013 *AJOG* [1,2,3,4]	All isolated bacteria are considered contamination; the uterus is still considered sterile.
2015	Mitchell CM et al., *AJOG* [5]	First multi-site 16S rRNA study of the female reproductive tract → proves the presence of microbial communities in the upper sections (incl. uterus).
2016	Fang RL et al., *AJTR* [7]	It clearly shows through 16S rRNA sequencing that the endometrium has its own microbiome, distinct from the vaginal one.
2016	Moreno I. et al., *AJOG* [6]	Connects endometrial microbiome with implantation success/failure in IVF—first clinical data for functional meaning.
2016	Verstraelen H. et al., *PeerJ* [8]	Deep sequencing on the endometrium in healthy women → confirms that the uterus is not sterile.
2017–2019	Several groups [12,13,14] (Chen. et al., 2017, *Nat. Commun.*, Kyono et al., 2019, *RMB,* Winters et al., 2019, *Sci. Rep.*)	Additional NGS studies → confirm domination of *Lactobacillus* in a “normal” profile and relationship to fertility.
2019–2022	Multi-omics approaches (metagenomics, metabolomics), Jean and all. 2019 *Infect. Microbes Dis.*, Boroń et al., 2022 *IJMS*, Bokulich et al., 2022 *PLoS Compute. Biol*., Jie et al., 2022 *GPB*, Chen et al., 2021 *Front. Cell Dev. Biol*. [15,16,17,18,19]	They reveal the functional role of microbes: estrogen metabolism, inflammation, and immune regulation.
2023–2025	Large cohort and meta-analyses (Bui et al., 2023 *Sci. Rep.*, Foteinidou et al., 2024 *AMH*, Su et al., 2024 *Reprod Biol Endocrinol*, Li et al., 2025 *Msystems*, Ye and D imitriadis 2025 *Biomolecules*) [20,21,22,23,24]	They emphasize the clinical significance of the LD (“*Lactobacillus*-dominant”) profile and create predictive models for IVF success.

**Table 2:** Research on the endometrial microbiome through different methods—advantages and disadvantages.

Method	Advantages	Restrictions	Suitable Applications
qPCR panels	Fast, sensitive, purposeful.	Limited to in advance selected taxa.	Clinical practice (EMMA test), validation of results.
16S rRNA sequencing	Cheap, widely used, suitable for ato taxonomic profile. Applicable to many types of samples and designs in research.	Limited resolution (up to genus/species), no reports of functional activity, and the need for control samples and standardization in 16S ampliconization. Requires knowledge of microbial community.	Basic studies on composition.
Shotgun metagenomics	High resolution, functional information, and discovery of rare microorganisms. No required knowledge for microbial community.	Expensive, complicated analysis, requires high-quality DNA.	In detailed profiling, multi-omics integrations.

**Table 3:** Microbiome profile at health vs. pathology.

Condition	Characteristic Taxa	Potentially Meaning
Healthy endometrium	*Lactobacillus crispatus*, *L. iners*	Maintenance on low pH, antimicrobial protection, and optimal implantation
Implantation failures	↑ *Gardnerella, Atopobium*, *Streptococcus*	Inflammation, impaired endometrial acceptance
Chronic endometritis	↑ *Enterococcus*, *Escherichia coli*	Persistent inflammation, risk of infertility
Endometriosis	Imbalance: ↑ *Streptococcus*, *Staphylococcus*	Potential contribution to a chronic inflammatory background

**Table 4:** Three-dimensional human endometrial models.

Characteristics	Spheroids	Organoids	Assembloids
Cellular composition	Single-type or mixed cells	Epithelial stem/progenitor cells	Complex of epithelium, stroma, and immune cells
Architecture	Simple aggregates	Gland-like 3D structures	Complex 3D systems with multicellular interaction
Physiological relevance	Medium	High	Very high (closer to in vivo)
Main applications	Adhesion, invasion	Receptivity, pathologies, and hormonal response	Integrative models of implantation and immune regulation
Limitations	Absence of complex structure	Absence of stromal and immune cells	Complex and resource-intensive, still a developing technology

**Table 5:** Oxidative stress, biomarkers, and effects on the female reproductive system.

Factor/Mechanism	Impact/Consequence	Conditions	Ref.
ROS	DNA damage, cell apoptosis, aging	Endometriosis, PCOS, infertility	[120]
Lipid peroxidation	Impaired membrane fluidity, cellular dysfunction	Endometriosis	[136]
Elevated cytokines, macrophages	Chronic inflammation, toxic to sperm/embryos	Endometriosis	[136]
Glycodilin + VEGF	Promotes the growth of ectopic endometrium	Endometriosis	[137]
Impaired iron metabolism	ROS generated by the Fenton reaction	Endometriosis	[138]
DNA damage (γH2AX, p16INK4A)	Cellular senescence, decreased implantation	Endometriosis	[139]
Low antioxidant activity (SOD, GPx)	Neutralization of ROS is difficult	Endometriosis, PCOS	[118]
Infections (e.g., *E. faecalis*)	Apoptosis and decreased receptivity of the endometrium	Infertility	[139]
Psychological stress	Increased ROS, hormonal imbalance	PCOS, Infertility	[140]
Severity of endometriosis	Proportional to OS level	Endometriosis	
Experimental therapies (antioxidants)	Limited effectiveness on conception	Mild endometriosis	

**Table 6:** Main compounds and enzymes associated with the antioxidant function of LAB.

Compound/Enzyme	Function	Availability/Role in LAB	Ref.
Glutathione (GSH)	Universal antioxidant, supports redox balance, protects the cells from stress	*Lc. lactis*, *L. fermentum ME-3*, *L. salivarius*, *L. reuteri*	[164,165,166,167,168]
Glutathione reductase (GshR)	Restores GSSG back to GSH, maintains active redox cycle	*L. plantarum*, *L. sanfranciscensis*	[169,170]
Glutathione synthetase/ligase	Synthesize glutathione from precursors	LAB use or bienzyme, or a bifunctional synthetic route	[171]
Cysteine (precursor)	Predecessor of GSH; alone by itself, there is antioxidant function	Supports growth and antioxidant protection at different strains (*L. sanfranciscensis*)	[166,170]
γ-glutamyl-cysteine	Precursor of glutathione	Participates in biosynthesis and redox balance	[171]
Thioredoxin reductase/ Thioredoxin	Protects enzymes like GAPDH, involved in redox reactions, important in peroxide stress	*L. plantarum WCFS1*, *B. bifidum*	[172,173,174]
Glutaredoxin system	Together with thioredoxin, the system supports the cytoplasm in reduced condition	General mechanism in many LAB	[171]
H_2_S and thiols	Products from the decomposition of cystine; act as reducing agents	*L. reuteri*—through ABC transporters and cystathionine γ-lyase	[171]

## Data Availability

No new data were created or analyzed in this study. Data sharing is not applicable to this article.
